# Prevalence, Incidence, and Recovery of Metabolic Dysfunction-associated Steatotic Liver Disease and Associations With Weight Loss and Lipid Reduction in a Chinese Community-based Cohort

**DOI:** 10.2188/jea.JE20240224

**Published:** 2025-04-05

**Authors:** Yurou Xu, Youyi Wang, Xiajing Yao, Qi Zhao, Bo Chen, Na Wang, Tiejun Zhang, Yonggen Jiang, Yiling Wu, Na He, Genming Zhao, Zhongxing Sun, Xing Liu

**Affiliations:** 1 The Key Laboratory of Public Health Safety of Ministry of Education, Department of Epidemiology, School of Public Health, Fudan University, Shanghai, China; 2 Songjiang District Center for Disease Control and Prevention, Shanghai, China

**Keywords:** metabolic dysfunction associated steatotic liver disease, prevalence, incidence, recovery, weight loss, cohort study

## Abstract

**Background:**

As the most common chronic liver disease worldwide, the natural history of metabolic dysfunction-associated steatotic liver disease (MASLD) in the general population is barely reported.

**Methods:**

The Shanghai Suburban Adult Cohort and Biobank study recruited 36,404 adults between 2016 and 2017, and followed up 25,085 participants between 2019 and 2023 in Songjiang District. A questionnaire survey was conducted using face-to-face interview, and physical examination and laboratory tests were conducted. MASLD was diagnosed using liver ultrasound and the cardiometabolic risk factors (CMRF).

**Results:**

A total of 36,122 and 21,831 participants met the criteria for baseline and follow-up analyses. The prevalence of MASLD at baseline was 36.8% overall, and 73.6% among those with a body mass index (BMI) over 28 kg/m^2^. After a median follow-up time of 4.26 years, the incidence density for MASLD was 8.4, and the recovery density was 11.4 per 100 person-years overall and was 20.0 and 8.4 per 100 person-years for those with baseline BMI over 28 kg/m^2^. Per 1 kg/m^2^ increase in baseline BMI was associated with a 15% increase in incidence (hazard ratio [HR] 1.15; 95% confidence interval [CI], 1.14–1.17) and an 8% decrease in recovery (HR 0.92; 95% CI, 0.90–0.93). From baseline to follow-up visit, participants who remained non-obese or remained normal cardiometabolic status always showed the lowest incidence and the highest recovery rate, followed by those with improved status.

**Conclusion:**

The prevalence and incidence of MASLD were high among Shanghai residents, and active recovery was also observed. Obesity was the most important risk factor, and weight loss and lipid level reduction were beneficial for preventing or reversing MASLD.

## INTRODUCTION

Metabolic dysfunction-associated steatotic liver disease (MASLD), formerly named as non-alcoholic fatty liver disease, is characterized by excessive accumulation of hepatic lipids associated with metabolic syndrome.^1^ Without intervention, its progression to metabolic dysfunction-associated steatohepatitis, cirrhosis, and liver cancer imposes significant health and economic burdens.^2^ Extensive hepatitis B virus vaccination efforts and successful drug development for hepatitis C virus have shifted MASLD to the most common and important chronic liver disease worldwide.^3^ MASLD is expected to be a leading cause of liver transplantation in Western countries by 2030.^4^ According to a meta-analysis in 2019, the global prevalence of MASLD was 30%, and that in Asia was 27%.^5^ As a developing country, China has also witnessed an increasing prevalence, incidence, and mortality of MASLD, which were even higher than those in other Asian regions.^6^ It is anticipated that China will have the highest number of patients with MASLD, estimated at 314 million by 2030, and liver-related deaths worldwide.^7^

Given the ongoing epidemic of MASLD, urgent action is needed to prevent and control MASLD in communities based on a better understanding of its epidemiology and natural history. Limited research has examined the incidence of MASLD in Chinese community. Furthermore, given its reversible nature at an early stage, the recovery rate is also an important measure for a better understanding of the natural history of the disease. However, little is known about the recovery rate and its correlation in community settings. MASLD is closely associated with obesity and metabolic syndromes. Urbanization and popularity of Western dietary patterns in China over recent decades have also set the stage for an epidemic of obesity and metabolic syndrome.^8^ Excess body fat, including higher body mass index (BMI) and visceral fat, is considered a primary risk factor for MASLD onset and development, and weight loss is a well-recognized therapeutic approach for MASLD.^9^ Abnormal blood lipids, particularly elevated levels of cholesterol, triglycerides, and low-density lipoprotein cholesterol, may disrupt lipid metabolism and accelerate fat deposition in the liver.^10^ However, the magnitude of the associations of overweight, obesity, and blood lipids with MASLD in the Chinese population remains unclear, and little is known about the effects of changes in body fat or blood lipids on MASLD incidence or recovery.

In this study, we utilized both baseline and follow-up data from a community-based cohort study to estimate the prevalence, incidence, and recovery rate of MASLD among residents in Shanghai. We also examined the risk factors for MASLD and the potential impact of changes in body fat, serum lipid levels, and other CMRFs on the condition to devise effective intervention strategies.

## METHODS

### Study design and participants

The population-based Shanghai Suburban Adult Cohort and Biobank study was designed to identify environmental, lifestyle and genetic risk factors for non-communicable chronic diseases.^11^ We used a multistage, stratified, clustered sampling method to select four communities in Songjiang district as study sites by economic level, population size, and availability of healthcare facilities. The baseline enrollment and survey were conducted from June 2016 to August 2017, and residents who were natives of the Shanghai municipality or who had been living in Shanghai for 5 years or longer were recruited. A total of 36,404 participants aged 20–74 years were enrolled at baseline. Follow-up visits were integrated into the annual Shanghai Senior Citizen Health Examination Program from 2019 to 2023. This program included a basic questionnaire survey, physical examination (including liver ultrasonography), and laboratory tests for biochemical markers. By August 2023, 25,085 participants were followed up, with a follow-up rate of 68.9%. The selection criteria are shown in eFigure 1, and 36,122 and 21,831 participants met the inclusion criteria for baseline and follow-up analyses, respectively. The study protocol was approved by the Ethical Committee of the School of Public Health, Fudan University (IRB approval number 2016-04-0586). Informed written consent was obtained from all participants before data collection.

### Data collection

Sociodemographic characteristics, personal and family history of chronic diseases, and lifestyle and dietary patterns were collected using a structured personal health questionnaire at baseline. Interviewers were staff of local community healthcare centers and were trained before conducting the face-to-face interviews. All participants enrolled in the study received physical examinations by licensed medical physicians at the local community healthcare centers at both baseline and follow-up visits, including anthropometric measurements (height, weight, waist circumference [WC], hip circumference, and resting blood pressure) and B mode ultrasound. Blood samples were collected at both baseline and follow-up, and common laboratory tests, including fasting plasma glucose, glycated hemoglobin, and serum lipids, were conducted.

### Diagnosis of MASLD and its incidence and recovery

Diagnosis of MASLD was based on evidence of hepatic steatosis on liver ultrasound, absence of any other cause of steatosis, and presence of one or more CMRFs.^1^ Liver ultrasound measurements were conducted both at baseline and during the follow-up assessments. Diagnosis of liver steatosis was defined as diffuse increased echogenicity of the liver parenchyma, with significant attenuation of image quickly within 4–5 cm of depth, reduced clarity of intrahepatic blood vessel structures, liver uniformly heterogeneous, and unclear liver texture. Other causes of steatosis, such as excessive alcohol consumption (>30 g/day for males and >20 g/day for females) and viral hepatitis, were assessed using a baseline questionnaire and examination. CMRF criteria included: BMI ≥23 kg/m^2^ or WC ≥80 cm in females and ≥94 cm in males; pre-diabetes (fasting plasma glucose of ≥5.6 mmol/L or hemoglobin A1c ≥5.7%), type 2 diabetes, or treatment for type 2 diabetes; blood pressure ≥130/85 mm Hg or receiving any antihypertensive treatment; plasma triglycerides (TG) ≥1.70 mmol/L or receiving any drug treatment; and plasma high-density lipoprotein cholesterol (HDL-C) ≤1.0 mmol/L in females and ≤1.3 mmol/L in males or receiving any drug treatment. As liver ultrasounds were repeated during follow-up visits, we focused on the first change in fatty liver status and did not consider subsequent changes for simplification in the current analysis. Incidence referred to the development of liver steatosis detected using ultrasound during follow-up in individuals without MASLD at baseline. Conversely, recovery from MASLD was defined as non-steatosis liver status observed during follow-up in individuals initially diagnosed with MASLD.

### Major covariates definition

BMI was defined as weight in kilograms divided by height in meters squared (kg/m^2^). BMI was classified into four categories: underweight (<18.5 kg/m^2^), normal (18.5–<24.0 kg/m^2^), overweight (24.0–<28.0 kg/m^2^), and obesity (≥28.0 kg/m^2^).^12^ The waist-to-hip ratio (WHR) was defined as the WC divided by the hip circumference. Participants with higher WC or WHR (WC ≥90 cm or WHR >0.90 for men and WC ≥85 cm or WHR >0.85 for women) were considered to have central obesity.^12^ Physical activity was quantified by calculating metabolic equivalent tasks (METs) for different activities (eTable 1), and was divided in four categories: <8.0, 8.0–<24.0, 24.0–40.0, and ≥40.0 MET-hours/day. Sedentary time was obtained by baseline questionnaire and was divided into three categories: 0–4, >4–8, and >8 hours/day. Smoking status was categorized as never, former, or current. Daily alcohol consumption was divided into four categories: 0, 0.1–10.0, 10.1–20.0, and 20.1–30.0 g/day. Chronic diseases including hypertension, diabetes, and hyperlipidemia were defined based on self-reported medical history, measurements or biochemical test results at baseline and follow-up, and medication history from electronic medical records.

### Statistical analysis

We calculated the prevalence, incidence density (ID), and recovery density (RD) of MASLD in the study population and across different sociodemographic characteristics and further stratified by sex. The person-years of incidence started from the date baseline survey for those at risk without MASLD at baseline, and it ended either at the date of the first ultrasound detection of liver steatosis during follow-up or at the date of the last follow-up visit (if liver steatosis has never been detected). For those with MASLD at baseline, recovery person-years were calculated from the date of the baseline survey to the date when liver steatosis was first undetected by ultrasound during follow-up or the date of the last follow-up visit (if liver steatosis was consistently detected). Multivariable logistic regression models were used to identify factors associated with the prevalence of MASLD with odds ratios (OR) and 95% confidence intervals (CI). Cox proportional hazard regression models were used to explore risk factors associated with the ID and RD of MASLD with hazard ratios (HR) and 95% CI. Both the logistic and Cox models were adjusted for sex, age group, education level, marital status, occupation, BMI, WC, WHR, physical activity, sedentary time, smoking status, alcohol intake, family history of fatty liver, hyperlipidemia, DM, and hypertension measured at baseline. Results from crude models without adjustments were presented in eTable 2, eTable 3, and eTable 4. When follow-up data were added, we compared the associations of changes from baseline to follow-up visit in BMI (remained normal, normal to overweight/obese, overweight/obese to normal, and remained overweight/obese), WC (remained normal, normal to central obesity, central obesity to normal, and remained central obesity), serum lipid levels (normal, normal to hyperlipemia, hyperlipemia to normal, and remained hyperlipemia), hyperglycemia change (normal, normal to hyperglycemia, hyperglycemia to normal, and remained hyperglycemia), and hypertension change (normal, normal to hypertension, hypertension to normal, and remained hypertension) with ID and RD of MASLD. We further adjusted for the documented use of hypolipidemic medication during the follow-up period when comparing the association between changes in serum lipid levels. Data were analyzed using SAS 9.4 (SAS Institute Inc., Cary, NC, USA). *P* values of less than 0.05 were considered to be statistically significant.

## RESULTS

### General characteristics of the study population

The sociodemographic characteristics of the study population are shown in Table 1. The 36,122 baseline participants had an average age of 56.4 (standard deviation [SD], 11.2) years, with 59.4% female, 52.9% with BMI ≥24.0 kg/m^2^, 28.9% with central obesity based on WC, and 56.7% based on WHR. Of the 21,831 followed up, the average age was 60.5 (SD, 10.0) years old, and the median follow-up time was 4.26 years.

**Table 1.** Demographic characteristics of baseline and follow-up populations among Songjiang residents in Shanghai, China, 2016–2017

Characteristics	Baseline population		Follow-up population		Follow-up population without MASLD at baseline		Follow-up population with MASLD at baseline	
			
	*N*	%	*N*	%	*N*	%	*N*	%
Total	36,122	100	21,831	100	13,052	100	8,779	100
Age, years								
18–39	3,510	9.8	777	3.6	523	4.0	254	2.9
40–49	4,514	12.5	1,349	6.2	863	6.6	486	5.5
50–59	11,841	32.8	6,225	28.5	3,473	26.6	2,752	31.4
60–69	12,605	34.9	10,434	47.8	6,172	47.3	4,262	48.6
≥70	3,652	10.1	3,046	14.0	2,021	15.5	1,025	11.7
Sex								
Male	14,671	40.6	7,962	36.5	5,015	38.4	2,947	33.6
Female	21,451	59.4	13,869	63.5	8,037	61.6	5,832	66.4
Education								
Illiterate or primary school	16,952	46.9	12,954	59.3	7,706	59.0	5,248	59.8
Middle school	12,775	35.4	6,459	29.6	3,843	29.4	2,616	29.8
High school	5,678	15.7	2,264	10.4	1,388	10.6	876	10.0
College or above	717	2.0	154	0.7	115	0.9	39	0.4
Marital status								
Married	33,546	92.9	20,171	92.4	11,983	91.8	8,188	93.3
Divorced/widowed	2,095	5.8	1,520	7.0	981	7.5	539	6.1
Single	481	1.3	140	0.6	88	0.7	52	0.6
Occupation								
Officer	1,254	3.5	592	2.7	370	2.8	222	2.5
Professional	2,787	7.7	1,403	6.4	854	6.5	549	6.3
Worker	8,285	22.9	4,506	20.6	2,645	20.3	1,861	21.2
Farmer	7,999	22.1	6,349	29.1	3,783	29.0	2,566	29.2
Other	15,797	43.7	8,981	41.1	5,400	41.4	3,581	40.8
BMI, kg/m^2^								
<18.5	931	2.6	459	2.1	449	3.5	10	0.1
18.5–<24.0	15,922	44.5	9,220	42.6	7,548	58.3	1,672	19.3
24.0–<28.0	14,104	39.4	8,860	41.0	4,241	32.8	4,619	53.2
≥28.0	4,835	13.5	3,091	14.3	705	5.5	2,386	27.5
Missing	330		201		109		92	
WC, cm								
Male <90/Female <85	25,385	71.1	14,598	67.6	10,534	81.6	4,064	46.9
Male ≥90/Female ≥85	10,336	28.9	6,983	32.4	2,376	18.4	4,607	53.1
Missing	401		250		142		108	
WHR								
Male ≤0.90/Female ≤0.85	12,944	43.3	6,671	37.3	5,159	48.0	1,512	21.1
Male >0.90/Female >0.85	16,966	56.7	11,234	62.7	5,581	52.0	5,653	78.9
Missing	6,212		3,926		2,312		1,614	
Smoking status								
Never	27,598	76.4	17,493	80.1	10,258	78.6	7,235	82.4
Former	1,353	3.8	866	4.0	567	4.3	299	3.4
Current	7,171	19.9	3,472	15.9	2,227	17.1	1,245	14.2
Alcohol intake, gram/day								
0	31,297	87.6	19,915	92.5	11,872	92.2	8,043	92.8
0.1–10.0	1,456	4.1	981	4.6	625	4.9	356	4.1
10.1–20.0	645	1.8	443	2.1	256	2.0	187	2.2
20.1–30.0	315	0.9	199	0.9	119	0.9	80	0.9
>30.0	2,003	5.6	—	—	—	—	—	—

BMI, body mass index; MASLD, metabolic-associated steatotic liver disease; WC, waist circumference; WHR, waist-to-hip ratio.

### Prevalence of MASLD at baseline

The prevalence of MASLD at baseline was 36.8% overall, 33.3% in males, and 39.2% in females (Table 2). Significant associations between MASLD and age differed according to sex. For males, the highest prevalence of 41.6% appeared in the age group 40–49 years and gradually decreased to 24.0% in those aged over 70 years. For females, the prevalence increased with age until 60–69 years (45.1%) and then decreased. The prevalence increased with higher BMI, with 16.6% for normal individuals and 73.6% for obese individuals. The participants with central obesity showed a higher prevalence of MASLD (64.0% vs 25.8% based on WC and 48.5% vs 21.1% based on WHR). Participants with a family history of fatty liver showed a higher prevalence than those without (48.3% vs 36.2%).

**Table 2.** Prevalence of MASLD and multivariate logistic regression for associations between characteristics with MASLD among Songjiang residents

Characteristics	Total (*N* = 36,122)		Male (*N* = 14,671)		Female (*N* = 21,451)	
		
	Prevalence, %	OR (95% CI)^**^	Prevalence, %	OR (95% CI)^*^	Prevalence, %	OR (95% CI)^*^
Total	36.8	—	33.3	—	39.2	1.15 (1.07–1.24)
Age, years						
18–39	26.7	1	40.9	1	18.2	1
40–49	34.1	1.13 (0.99–1.28)	41.6	0.87 (0.71–1.07)	29.7	1.45 (1.22–1.73)
50–59	42.0	1.25 (1.11–1.41)	38.4	0.74 (0.62–0.90)	44.1	1.91 (1.62–2.26)
60–69	37.4	0.85 (0.74–0.97)	28.1	0.45 (0.37–0.55)	45.1	1.48 (1.24–1.78)
≥70	31.0	0.50 (0.43–0.58)	24.0	0.30 (0.24–0.38)	37.1	0.84 (0.68–1.03)
Education						
Illiterate or primary school	38.0	1	27.9	1	43.1	1
Middle school	37.5	1.13 (1.06–1.21)	36.5	1.18 (1.06–1.32)	38.3	1.14 (1.05–1.24)
High school	33.4	1.08 (0.99–1.19)	37.5	1.05 (0.90–1.21)	29.7	1.16 (1.02–1.32)
College or above	25.7	1.07 (0.85–1.35)	35.6	0.92 (0.66–1.29)	18.4	1.24 (0.89–1.74)
Marital status						
Married	37.1	1	33.6	1	39.7	1
Divorced/Widowed	34.1	0.80 (0.71–0.90)	27.2	0.93 (0.72–1.22)	36.2	0.75 (0.66–0.85)
Single	26.6	0.93 (0.71–1.21)	31.2	0.79 (0.56–1.12)	20.3	0.99 (0.64–1.55)
Occupation						
Officer	34.3	1	37.6	1	30.2	1
Professional	35.6	1.14 (0.95–1.35)	35.4	1.13 (0.90–1.44)	35.8	1.07 (0.82–1.40)
Worker	38.0	1.08 (0.92–1.27)	34.8	1.09 (0.87–1.36)	39.9	1.06 (0.84–1.33)
Farmer	37.7	1.03 (0.88–1.21)	28.0	1.09 (0.86–1.38)	42.7	0.96 (0.76–1.22)
Other	36.2	1.01 (0.87–1.18)	33.8	1.01 (0.81–1.24)	37.8	1.00 (0.80–1.25)
BMI, kg/m^2^						
<18.5	1.4	0.08 (0.05–0.15)	1.8	0.15 (0.06–0.37)	1.2	0.07 (0.03–0.14)
18.5–<24.0	16.6	1	12.9	1	18.8	1
24.0–<28.0	49.4	4.13 (3.89–4.39)	42.2	4.15 (3.73–4.61)	55.3	4.12 (3.82–4.44)
≥28.0	73.6	8.70 (7.89–9.61)	67.0	8.22 (6.95–9.71)	78.5	9.01 (7.96–10.19)
WC, cm						
Male <90/Female <85	25.8	1	22.7	1	27.9	1
Male ≥90/Female ≥85	64.0	2.22 (2.08–2.38)	59.8	2.77 (2.48–3.09)	66.8	1.90 (1.75–2.07)
WHR						
Male ≤0.90/Female ≤0.85	21.1	1	18.8	1	23.0	1
Male >0.90/Female >0.85	48.5	2.26 (2.13–2.41)	42.5	2.28 (2.07–2.52)	52.1	2.17 (2.00–2.35)
Smoking status						
Never	38.8	1	37.3	1	39.2	1
Former	30.8	0.90 (0.76–1.06)	30.8	0.98 (0.83–1.16)	25.0	0.50 (0.11–2.18)
Current	30.4	0.93 (0.85–1.02)	30.3	0.91 (0.83–1.00)	48.9	1.89 (0.89–4.02)
Alcohol intake, g/day						
0	38.9	1	38.0	1	39.3	1
0.1–10.0	38.6	0.97 (0.85–1.11)	38.6	1.00 (0.86–1.15)	38.7	0.97 (0.62–1.52)
10.1–20.0	43.1	1.14 (0.94–1.39)	43.5	1.19 (0.98–1.46)	29.4	0.60 (0.16–2.22)
20.1–30.0	43.2	1.06 (0.80–1.40)	43.9	1.07 (0.81–1.42)	0	—
Family history of fatty liver						
No	36.2	1	32.7	1	38.7	1
Yes	48.3	1.59 (1.41–1.79)	48.0	1.77 (1.41–2.23)	48.4	1.52 (1.32–1.75)
History of hepatitis						
No	37.0	1	33.5	1	39.3	1
Yes	32.0	0.78 (0.66–0.92)	28.9	0.78 (0.62–0.99)	35.5	0.78 (0.62–0.97)

BMI, body mass index; MASLD, metabolic-associated steatotic liver disease; WC, waist circumference; WHR, waist-to-hip ratio.

^*^Multivariable logistic regression model adjusted for age, education, marital status, occupation, BMI, WC (not adjusted when WHR was included), physical activity, sedentary time, smoking, drinking, family history of fatty liver, hyperlipidemia, DM, and hypertension.

^**^Model further adjusted for sex.

—: Regression analyses were not conducted for categories of smoking status and alcohol intake in females due to insufficient sample size.

### Incidence density and recovery density of MASLD at follow-up

The median follow-up time was 4.33 years with a total of 56,551.78 person-years for estimating incidence density. The ID of MASLD was 8.4/100 person-years overall and was 7.9 for males and 8.7 for females (Table 3). The ID increased along with the increasing age in females, while decreased with age in males. Higher baseline BMI was significantly associated with increased ID. Participants who were obese at baseline showed nearly three times the risk of normal BMI group (HR 2.83; 95% CI, 2.51–3.19). Per 1 kg/m^2^ increase in baseline BMI (HR 1.15; 95% CI, 1.14–1.17), per 1 cm increase in WC (HR 1.04; 95% CI, 1.03–1.04) and per 0.05 increase in WHR (HR 1.13; 95% CI, 1.09–1.16) were all positively associated with MASLD incidence.

**Table 3.** Incidence density of MASLD and Cox proportional hazard regression of risk factors for MASLD incidence in Songjiang district

Characteristics	Total (*N* = 13,052)		Male (*N* = 5,015)		Female (*N* = 8,037)	
	
	ID, per 100 PYs	HR (95% CI)^***^	ID, per 100 PYs	HR (95% CI)^**^	ID, per 100 PYs	HR (95% CI)^*^
Total	8.4	—	7.9	—	8.7	1.08 (0.99–1.18)
Age, years						
18–39	5.1	1	9.7	1	3.3	1
40–49	6.0	0.93 (0.70–1.23)	8.4	0.67 (0.44–1.04)	5.3	1.28 (0.87–1.89)
50–59	7.0	0.83 (0.65–1.07)	6.4	0.42 (0.29–0.59)	7.3	1.32 (0.93–1.89)
60–69	9.8	1.39 (1.08–1.78)	8.6	0.76 (0.54–1.08)	10.7	2.11 (1.47–3.03)
≥70	8.3	1.02 (0.79–1.32)	7.6	0.59 (0.41–0.86)	8.9	1.50 (1.03–2.18)
Education						
Illiterate or primary school	9.2	1	8.1	1	9.8	1
Middle school	7.4	1.00 (0.93–1.09)	7.9	1.09 (0.97–1.23)	7.0	0.95 (0.85–1.06)
High school or above	6.9	0.95 (0.84–1.08)	7.7	0.98 (0.82–1.17)	6.1	0.93 (0.78–1.12)
Marital status						
Married	8.4	1	7.9	1	8.7	1
Divorced/Widowed/Single	8.5	0.85 (0.76–0.96)	8.0	0.93 (0.73–1.19)	8.7	0.83 (0.73–0.95)
Occupation						
Officer	7.7	1	7.9	1	7.4	1
Professional	7.9	1.10 (0.87–1.40)	7.8	1.13 (0.83–1.54)	7.9	1.06 (0.73–1.54)
Worker	8.3	1.24 (1.00–1.54)	8.8	1.51 (1.13–2.02)	8.0	1.02 (0.74–1.42)
Farmer	9.9	1.31 (1.05–1.62)	8.7	1.45 (1.08–1.94)	10.5	1.15 (0.83–1.59)
Other	7.6	0.97 (0.79–1.20)	7.1	1.02 (0.77–1.35)	7.9	0.89 (0.65–1.22)
BMI, kg/m^2^						
<18.5	1.2	0.19 (0.12–0.30)	1.3	0.15 (0.06–0.41)	1.1	0.21 (0.13–0.35)
18.5–<24.0	5.8	1	5.1	1	6.1	1
24.0–<28.0	13.1	2.05 (1.91–2.20)	11.8	2.17 (1.94–2.44)	14.2	1.98 (1.82–2.16)
≥28.0	20.0	2.83 (2.51–3.19)	19.3	3.32 (2.70–4.08)	20.5	2.65 (2.28–3.07)
per 1 kg/m^2^ increase		1.15 (1.14–1.17)		1.17 (1.15–1.19)		1.15 (1.13–1.16)
WC, cm						
Male <90/Female <85	7.0	1	6.9	1	7.1	1
Male ≥90/Female ≥85	15.6	1.33 (1.23–1.44)	14.4	1.17 (1.02–1.35)	16.2	1.40 (1.28–1.54)
per 1 cm increase		1.04 (1.03–1.04)		1.03 (1.02–1.04)		1.03 (1.03–1.04)
WHR						
Male ≤0.90/Female ≤0.85	6.7	1	6.7	1	6.6	1
Male >0.90/Female >0.85	11.8	1.26 (1.17–1.35)	11.2	1.13 (1.01–1.26)	12.1	1.34 (1.22–1.47)
per 0.05 increase		1.13 (1.09–1.16)		1.10 (1.04–1.16)		1.14 (1.10–1.18)
Smoking status						
Never	8.7	1	8.6	1	8.7	1
Former	7.0	0.65 (0.54–0.79)	6.9	0.66 (0.55–0.80)	9.9	—
Current	7.6	0.89 (0.80–0.99)	7.6	0.90 (0.81–1.00)	5.1	—
Alcohol intake, gram/day						
0	8.5	1	8.0	1	8.7	1
0.1–10.0	7.5	0.87 (0.75–1.02)	7.4	0.88 (0.75–1.04)	9.8	0.89 (0.49–1.61)
10.1–20.0	7.6	0.90 (0.72–1.13)	7.6	0.90 (0.71–1.13)	10.5	1.58 (0.22–1.28)
20.1–30.0	8.1	0.91 (0.67–1.24)	8.1	0.92 (0.68–1.25)	—	—
Family history of fatty liver						
No	8.4	1	7.9	1	8.8	1
Yes	8.1	0.93 (0.78–1.10)	7.7	1.05 (0.75–1.48)	8.2	0.91 (0.74–1.10)

BMI, body mass index; CI, confidence interval; ID, incidence density; MASLD, metabolic-associated steatotic liver disease; PYs, person-years; WC, waist circumference; WHR, waist-to-hip ratio.

^*^Cox proportional hazard regression model adjusted for age, education, marital status, occupation, BMI, WC (not adjusted when WHR was included), physical activity, sedentary time, drinking, family history of fatty liver, baseline hyperlipidemia, DM, and hypertension.

^**^Model further adjusted for smoking.

^***^Model further adjusted for sex.

—: Regression analyses were not conducted for categories of smoking status and alcohol intake in females due to insufficient sample size.

The median follow-up time was 4.15 years, with a total of 36,443.46 person-years for estimating the recovery rate. The RD of MASLD was 11.4/100 person-years overall and was 12.4 for males and 10.9 for females (Table 4). Compared with the lowest group, those aged 50–59 years had the lowest RD (9.0/100 person-years), while those aged 70 years and above had the highest RD (16.0/100 person-years), and a similar trend was observed across sex. Per 1 kg/m^2^ increase in BMI (HR 0.92; 95% CI, 0.90–0.93), per 1 cm increase in WC (HR 0.973; 95% CI, 0.965–0.981), and per 0.05 increase in WHR (HR 0.89; 95% CI, 0.86–0.92) were all inversely associated with MASLD recovery. Participants with family history of fatty liver had a lower rate of recovery (HR 0.82; 95% CI, 0.70–0.96). Results of sensitivity analyses did not show significant changes in both ID and RD estimates when further adjusted for chronic diseases (data not shown).

**Table 4.** Recovery density of MASLD and Cox proportional hazard regression of risk factors for MASLD recovery in Songjiang district

Characteristics	Total (*N* = 8,779)		Male (*N* = 2,947)		Female (*N* = 5,832)	
	
	RD, Per 100 PYs	HR (95% CI)^***^	RD, Per 100 PYs	HR (95% CI)^**^	RD, Per 100 PYs	HR (95% CI)^*^
Total	11.4	—	12.4	—	10.9	0.83 (0.76–0.91)
Age, years						
18–39	10.8	1	10.1	1	11.7	1
40–49	9.9	0.87 (0.67–1.15)	10.4	1.03 (0.69–1.54)	9.6	0.70 (0.48–1.03)
50–59	9.0	0.67 (0.53–0.85)	8.8	0.70 (0.50–0.98)	9.0	0.57 (0.40–0.80)
60–69	12.3	1.08 (0.86–1.38)	13.8	1.37 (0.98–1.91)	11.6	0.85 (0.60–1.20)
≥70	16.0	1.59 (1.24–2.04)	19.1	2.10 (1.47–2.99)	14.5	1.19 (0.83–1.70)
Education						
Illiterate or primary school	12.0	1	14.3	1	11.4	1
Middle school	10.5	0.94 (0.87–1.02)	11.0	0.93 (0.82–1.05)	10.1	0.97 (0.87–1.07)
High school or above	10.4	0.89 (0.79–1.01)	11.3	0.97 (0.81–1.16)	9.4	0.82 (0.69–0.99)
Marital status						
Married	11.2	1	12.3	1	10.7	1
Divorced/Widowed/Single	13.9	1.19 (1.06–1.35)	14.8	1.20 (0.93–1.56)	13.6	1.19 (1.04–1.36)
Occupation						
Officer	11.9	1	12.9	1	11.9	1
Professional	10.2	0.83 (0.66–1.06)	11.8	0.96 (0.71–1.30)	7.7	0.63 (0.42–0.95)
Worker	10.5	0.98 (0.79–1.21)	11.1	1.02 (0.76–1.35)	10.3	0.84 (0.60–1.18)
Farmer	12.5	1.08 (0.87–1.33)	14.8	1.17 (0.88–1.56)	11.9	0.91 (0.65–1.28)
Other	11.2	1.07 (0.87–1.32)	12.1	1.20 (0.92–1.57)	10.8	0.89 (0.64–1.24)
BMI, kg/m^2^						
<18.5	25.7	2.12 (1.01–4.47)	55.4	4.04 (1.28–12.77)	18.3	1.41 (0.53–3.77)
18.5–<24.0	15.5	1	18.7	1	14.5	1
24.0–<28.0	11.7	0.74 (0.68–0.80)	12.9	0.66 (0.57–0.77)	11.0	0.77 (0.70–0.85)
≥28.0	8.4	0.57 (0.51–0.64)	8.8	0.47 (0.39–0.58)	8.2	0.62 (0.55–0.71)
per 1 kg/m^2^ increase		0.92 (0.90–0.93)		0.90 (0.88–0.92)		0.92 (0.91–0.94)
WC, cm						
Male <90/Female <85	13.7	1	15.2	1	13.0	1
Male ≥90/Female ≥85	9.5	0.75 (0.70–0.81)	10.2	0.77 (0.68–0.87)	9.2	0.75 (0.68–0.82)
per 1 cm increase		0.97 (0.97–0.98)		0.97 (0.96–0.98)		0.97 (0.97–0.98)
WHR						
Male ≤0.90/Female ≤0.85	14.3	1	15.5	1	13.5	1
Male >0.90/Female >0.85	11.1	0.78 (0.72–0.85)	12.1	0.82 (0.72–0.94)	10.7	0.76 (0.68–0.85)
per 0.05 increase		0.89 (0.86, 0.92)		0.90 (0.84–0.96)		0.89 (0.86–0.93)
Smoking status						
Never	11.2	1	12.3	1	10.9	—
Former	14.5	1.07 (0.89–1.28)	14.5	1.04 (0.87–1.25)	15.9	—
Current	12.0	1.04 (0.93–1.16)	12.1	1.05 (0.94–1.18)	7.9	—
Alcohol intake, gram/day						
0	11.4	1	12.8	1	10.9	—
0.1–10.0	10.8	0.89 (0.75–1.05)	11.1	0.94 (0.78–1.12)	6.9	—
10.1–20.0	11.1	0.88 (0.70–1.12)	10.9	0.85 (0.66–1.08)	21.9	—
20.1–30.0	11.7	0.98 (0.71–1.36)	11.7	1.01 (0.73–1.41)	—	—
Family history of fatty liver						
No	11.6	1	12.5	1	11.1	1
Yes	9.0	0.82 (0.70–0.96)	10.0	0.76 (0.56–1.03)	8.7	0.85 (0.71–1.01)

BMI, body mass index; CI, confidence interval; MASLD, metabolic-associated steatotic liver disease; RD, recovery density; WC, waist circumference; WHR, waist-to-hip ratio.

^*^Cox proportional hazard regression model adjusted for age, education, marital status, occupation, BMI, WC (not adjusted when WHR was included), physical activity, sedentary time, family history of fatty liver, baseline hyperlipidemia, DM, and hypertension.

^**^Model further adjusted for smoking and drinking.

^***^Model further adjusted for sex.

—: Regression analyses were not conducted for categories of smoking status and alcohol intake in females due to insufficient sample size.

### Incidence and recovery density of MASLD with changes in BMI, WC, serum lipid levels, hyperglycemia, and hypertension

**Figure 1.**
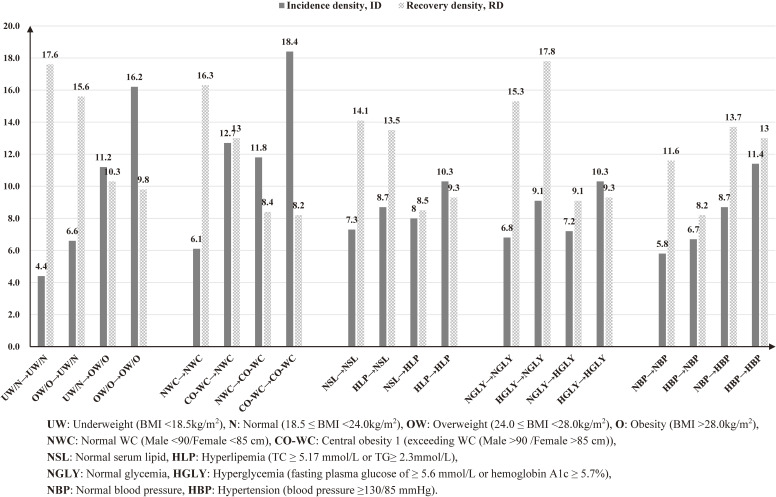
The incidence and recovery density of MASLD with changes of BMI, WC and serum lipid from baseline to follow-up visit

Participants who remained overweight/obese had the highest ID (16.2/100 person-years), while those who remained underweight/normal had the lowest ID (4.4/100 person-years) (Figure 1). Those who remained underweight/normal had the highest RD (17.6/100 person-years), followed with those who lost weight from overweight/obese to underweight/normal (15.6/100 person-years), whereas those who remained overweight/obese had the lowest RD (9.8 per 100 person-years). Similar results were observed for the changes in WC. Participants with remaining hyperlipidemia had the highest ID (10.3 per 100 person-years), and those who changed from a normal lipid level to hyperlipemia had the lowest RD (8.5 per 100 person-years). Both the remaining hyperglycemia group (10.3 per 100 person-years) and the remaining hypertension group (11.4 per 100 person-years) had the highest ID, compared with the remain normal groups. Similar results were also found for different sex, and sensitivity analyses did not show significant changes after adjusting for the history of hyperlipidemia, diabetes, hypertension, and documented use of medication (Table 5).

**Table 5.** The incidence and recovery density of MASLD with changes of BMI, WC, and serum lipid from baseline to follow-up visit

	Follow-up population without MASLD at baseline						Follow-up population with MASLD at baseline					
	
	Total (*N* = 13,052)		Male (*N* = 5,015)		Female (*N* = 8,037)		Total (*N* = 8,779)		Male (*N* = 2,947)		Female (*N* = 5,832)	
	
	ID/100 PY	HR (95% CI)^***^	ID/100 PY	HR (95% CI)^**^	ID/100 PY	HR (95% CI)^*^	RD/100 PY	HR (95% CI)^***^	RD/100 PY	HR (95% CI)^**^	RD/100 PY	HR (95% CI)^*^
**BMI change**												
Remain normal	4.4	1	3.8	1	4.8	1	17.6	1	21.3	1	16.4	1
Obese/overweight to normal	6.6	1.04 (0.90–1.19)	5.6	1.10 (0.88–1.39)	7.5	1.00 (0.83–1.20)	15.6	0.98 (0.87–1.10)	18.6	0.96 (0.78–1.19)	14.2	0.98 (0.84–1.13)
Normal to obese/overweight	11.2	2.13 (1.92–2.36)	11.1	2.45 (2.06–2.92)	11.2	1.97 (1.74–2.25)	10.3	0.59 (0.50–0.69)	12.7	0.59 (0.44–0.78)	9.5	0.58 (0.48–0.71)
Remain obese/overweight	16.2	2.40 (2.17–2.66)	14.9	2.69 (2.28–3.19)	17.3	2.25 (1.98–2.57)	9.8	0.73 (0.66–0.82)	10.6	0.65 (0.53–0.80)	9.4	0.78 (0.68–0.89)
**WC change**												
Remain normal	6.1	1	6.1	1	6.2	1	16.3	1	17.4	1	15.7	1
CO–WC to normal	12.7	1.44 (1.30–1.59)	10.9	1.23 (1.03–1.46)	13.7	1.52 (1.34–1.72)	13.0	0.78 (0.71–0.86)	13.3	0.79 (0.68–0.92)	12.9	0.79 (0.70–0.89)
Normal to CO–WC	11.8	1.46 (1.35–1.59)	12.0	1.53 (1.34–1.74)	11.6	1.44 (1.29–1.60)	8.4	0.50 (0.45–0.56)	9.0	0.49 (0.40–0.61)	8.2	0.51 (0.45–0.58)
Remain CO–WC	18.4	1.62 (1.48–1.78)	18.2	1.64 (1.40–1.92)	18.5	1.59 (1.41–1.79)	8.2	0.54 (0.50–0.60)	8.5	0.58 (0.49–0.68)	8.1	0.53 (0.47–0.60)
**Serum lipid change**												
Remain normal	7.3	1	7.1	1	7.5	1	14.1	1	16.0	1	12.8	1
Hyperlipemia to normal	8.7	1.09 (0.99–1.20)	7.2	1.05 (0.90–1.23)	9.6	1.09 (0.96–1.23)	13.5	0.90 (0.83–0.99)	13.7	0.84 (0.73–0.97)	13.3	0.97 (0.87–1.08)
Normal to hyperlipemia	8.0	1.13 (1.03–1.24)	8.3	1.29 (1.11–1.50)	7.9	1.03 (0.92–1.16)	8.5	0.57 (0.51–0.64)	8.7	0.50 (0.41–0.61)	8.5	0.62 (0.54–0.72)
Remain hyperlipemia	10.3	1.40 (1.31–1.51)	10.7	1.60 (1.42–1.80)	10.2	1.30 (1.19–1.42)	9.3	0.65 (0.60–0.70)	8.7	0.57 (0.49–0.65)	9.6	0.71 (0.64–0.78)
**Hyperglycemia change**												
Remain normal	6.8	1	6.3	1	7.1	1	15.3	1	16.4	1	14.7	1
Hyperglycemia to normal	9.1	1.15 (1.05–1.26)	8.8	1.09 (0.93–1.28)	9.2	1.18 (1.05–1.33)	17.8	1.17 (1.06–1.29)	19.8	1.17 (1.00–1.38)	17.0	1.17 (1.04–1.32)
Normal to Hyperglycemia	7.2	1.02 (0.92–1.13)	6.7	1.04 (0.88–1.22)	7.5	1.01 (0.89–1.15)	9.1	0.56 (0.50–0.63)	9.5	0.53 (0.44–0.64)	8.9	0.58 (0.51–0.67)
Remain Hyperglycemia	10.3	1.33 (1.23–1.44)	9.7	1.28 (1.12–1.46)	10.8	1.35 (1.22–1.48)	9.3	0.59 (0.54–0.64)	10.3	0.58 (0.50–0.66)	8.9	0.60 (0.54–0.66)
**Hypertension change**												
Remain normal	5.8	1	5.6	1	5.9	1	11.6	1	12.3	1	11.2	1
Hypertension to normal	6.7	1.03 (0.92–1.15)	6.8	1.13 (0.95–1.35)	6.7	0.95 (0.83–1.10)	8.2	0.70 (0.64–0.78)	8.9	0.75 (0.64–0.89)	7.8	0.69 (0.60–0.78)
Normal to Hypertension	8.7	1.09 (0.98–1.22)	8.0	1.00 (0.84–1.20)	9.2	1.13 (0.99–1.29)	13.7	1.38 (1.23–1.55)	16.5	1.56 (1.29–1.89)	12.4	1.29 (1.11–1.49)
Remain Hypertension	11.4	1.23 (1.12–1.34)	10.5	1.21 (1.04–1.40)	11.9	1.22 (1.09–1.37)	13.0	1.34 (1.22–1.47)	14.5	1.48 (1.27–1.72)	12.5	1.29 (1.15–1.45)

BMI, body mass index; CI, confidence interval; ID, incidence density; RD, recovery density; WC, waist circumference; CO–WC, Central obesity 1 (exceeding WC (Male >90/Female >85 cm)).

Hyperglycemia was diagnosed as fasting plasma glucose of ≥5.6 mmol/L or hemoglobin A1c ≥5.7%, and hypertension was diagnosed as blood pressure ≥130/85 mm Hg.

Note: ^*^Cox proportional hazard regression model adjusted for age, education, marital status, occupation, BMI, WC, physical activity, sedentary time, family history of fatty liver, history of hypolipidemic medication use, baseline hyperlipidemia, DM, and hypertension. ^**^Model further adjusted for smoking and drinking. ^***^Model further adjusted for sex.

## DISCUSSION

To our knowledge, this is the first community-based large-scale cohort study to report the prevalence, incidence, and recovery rates of MASLD and its correlates in the Chinese population. We observed a prevalence of 36.8%, an incidence of 8.4 cases, and a recovery rate of 11.4 cases per 100 person-years for MASLD among Shanghai residents. Age and sex heterogeneity were observed in the progression of MASLD, and obesity was found to be the most important risk factor. The participants who maintained normal weight and lipid levels showed the highest recovery rates from MASLD. Those with weight loss and reduced serum lipid levels showed slightly lower recovery, while those who became obese or developed hyperlipemia were less likely to recover when compared with those who remained normal. These findings revealed the disease burden and prevention targets for the currently most common chronic liver disease.

Previous studies indicated that the gross domestic product was positively correlated with the prevalence of MASLD in the past 20 years in China.^13^ Shanghai, as a representative of most developed cities in China, had a higher MASLD prevalence than other cities.^14^ When compared with developed areas, the MASLD prevalence in Shanghai was close to that in American adults (37.1%)^15^ and Korean adults (38.0%),^16^ while throughout the world it was considerably higher than in North America (35.3%), South America (35.7%), Europe (30.8%) and Africa (28.2%) according to a meta-analysis in 2021.^17^ Few studies reported the incidence of MASLD in the community-based general population. The incidence in our study was not only significantly higher than that in the Kailuan workers cohort in Hebei, China (4.2 per 100 person-years),^18^ but also higher than that in the health check-up study in Taiwan, China (7.0 per 100 person-years).^19^ The participants of the Kailuan were younger manual labor workers, which might explain the lower incidence. Even fewer studies had examined the recovery of MASLD in community, with limited research based on clinical trials or case-control studies.^20^ We first examined the natural progression and recovery of MASLD in detail based on this community cohort. We were thrilled to see that residents recover from MASLD actively with a rate of 11.4 cases per 100 person-years without special intervention. These results provide essential data on the MASLD epidemic and suggest potential intervention strategies. Unlike other chronic diseases, liver steatosis is dynamic, actively occurring and recovering. This study offers insights for future research, and further surveillance with advanced diagnostic methods is needed to study the epidemiology and natural history of MASLD in Shanghai and in other areas.

It was interesting to find that not only men and women showed different disease frequency, but sex acted as an effect-measure modifier for associations between other risk factors and MASLD as well. Females had higher incidence and lower recovery rate than males, and the association with age varied significantly between sex. With age increase, the prevalence and incidence increased in women while decrease in men, which is consistent with recent research.^21^ The underlying reasons for the sex difference need to be explored, but there were some explanations from both social and biological perspectives. In China, men of working age tend to engage in more social activities, leading to irregular diet, overeating, and alcohol drinking. Chinese women shoulder both work and household responsibilities, so they tend to be more physically active due to increased housework. After retirement, as their lifestyles become more relaxed, the risk of MASLD reached its peak. Biologically, women of reproductive age generally have a lower risk of MASLD compared to men, potentially due to the protective effect of estrogen. Studies using ovariectomized animal models have indicated a causal link between estrogen deficiency and an increased susceptibility to fatty liver and its development.^22^ However, this protective effect of estrogen may diminish after menopause, accompanied by subclinical disruptions in metabolic parameters.^23^ In future studies, more detailed examinations of sex difference are warranted for a better understanding of the underlying mechanisms in MASLD etiology.

The present study found that higher BMI, WC, and WHR were associated with a higher risk of MASLD, and with decreased possibility of MASLD recovery. People with BMI over 28 kg/m^2^ had nearly four times the incidence of MASLD than those with BMI of 18.5–<24.0 kg/m^2^. The findings were consistent with a meta-analysis in 2016, which showed a 3.5-fold higher risk of MASLD in obese individuals and a dose-dependent association between BMI and MASLD risk (per 1 kg/m^2^ increment in BMI: RR 1.20; 95% CI, 1.14–1.26).^24^ The rapid economic growth and urbanization in China have led to a notable shift in lifestyle and a rise in obesity rates among the population.^25^ In the present study, there were 58.4% of men being overweight with BMI ≥24 kg/m^2^ compared to 49.1% of women at baseline. The BMI variation between sex was less obvious than from a study of 15.8 million Chinese adults, which indicated 41.1% of men and 27.7% of women with BMI ≥24 kg/m^2^.^26^ The higher prevalence of overweight overall and in women in the present cohort might due to higher level of economic development in Shanghai and older age in our study population, with men’s prevalence of overweight decrease with aging, while women’s increase. Obesity, known as a consequence of a prolonged positive energy balance driven by biological, environmental, and social factors, is undoubtedly the foremost risk factor for MASLD.^27^ The imbalance results in the accumulation of excess lipids in adipose tissue and the liver, triggering chronic subclinical inflammation and insulin resistance, ultimately progressing to MASLD. Moreover, studies have found that obese individuals with MASLD tend to have higher fibrosis and MASLD activity scores, leading to a worse prognosis.^28^

Our further findings about the potential benefits of improvement in body fat and blood lipids to the regression of MASLD were also consistent with previous studies. Although those who maintained normal weight and lipid levels had the highest recovery rates, mainly due to an overall better health status, the weight-loss and lipid-lowering groups had comparable incidence and recovery rates, both of which were much better than the remaining-abnormal group. Weight loss is a well-recognized low-cost treatment for MASLD. In children and young adults, biomarkers of MASLD did improve following weight loss secondary to lifestyle modifications.^29^ For obese patients with MASLD, a weight loss of 3–5% can improve liver steatosis, and 7–10% can further improve liver enzymological and histological abnormalities.^4^ Besides, several trials have demonstrated that reducing body lipid levels could lower liver enzymes in the blood, enhance insulin sensitivity, and exacerbate the pathological progression of MASLD.^30^ Therefore, given the rising prevalence of obesity, it is imperative to promote lifestyle changes that prevent and reduce obesity and MASLD before the burden of end-stage liver diseases or other severe consequences of comorbidities become unavoidable. Our finding also showed potential effects of hyperglycemia and hypertension changes. While there may be an effect of medication control, changes in these CMRFs status are also factors of concern in the development and recovery from MASLD.

Our findings were strengthened by a comprehensive inclusion of variables and the prospectively collected data, thus reducing the probability for recall bias. Furthermore, we obtained data about the disease outcome and prescription of medication from the electric medical system, which allowed us to further adjust potential confounding. Several limitations of this study warrant acknowledgment. First, we diagnosed MASLD based on non-invasive B-ultrasound for fatty liver instead of histologic proof, such as biopsy, which may carry a risk of overdiagnosis. While biopsy or transient-elastography technology may provide a more accurate diagnosis, ultrasound is the most feasible way in large-scale community-based studies among healthy residents with adequate reliability and accuracy. Second, since the lifestyle factors were collected using self-report questionnaire, misclassification was inevitable. Third, due to lack of hip assessment at follow-up visits, we could not examine the association between MASLD and the changes in WHR. Last, we could not assess the severity of MASLD in the present study. Though we conducted thorough physical examinations administered by experienced and well-trained medical professionals, it’s worth noting that assessing fatty liver severity through ultrasonography can be subjective, and diagnosis may vary among healthcare providers. More rigorous prospective study designs are required in the future to explore the natural history of MASLD.

In conclusion, the prevalence and incidence of MASLD were high in the Chinese community, and active recovery from MASLD was also observed. Overweight, obesity, and central obesity were the most important predictors of risk of MASLD. Weight loss and reduced serum lipid showed significant associations with lower incidence and higher recovery rate of MASLD. Encouraging lifestyle modifications to prevent and mitigate obesity and MASLD is essential at its early reversible stage.

## SUPPLEMENTARY MATERIAL

eFigure 1. Flow chart of the study participants

eTable 1. Physical activity types, intensity categories, MET values and codes

eTable 2. Prevalence of NAFLD and univariate logistic regression for associations between demographic and behavioral characteristics with NAFLD among community residents in Shanghai, China

eTable 3. Incidence density of NAFLD and univariate Cox proportional hazard regression of risk factors for NAFLD incidence in Songjiang district

eTable 4. Recovery density of NAFLD and univariate Cox proportional hazard regression of risk factors for NAFLD recovery in Songjiang district
