# Supplementary material for: Prevalence, Incidence, and Recovery of Metabolic Dysfunction-associated Steatotic Liver Disease and Associations With Weight Loss and Lipid Reduction in a Chinese Community-based Cohort
Source: J Epidemiol. 2025 Apr 5;35(4):195–205. doi: 10.2188/jea.JE20240224 (PMC11882351; doi:10.2188/jea.JE20240224)
Supplement: Supplementary file 1 [file je-35-195-s001.pdf]

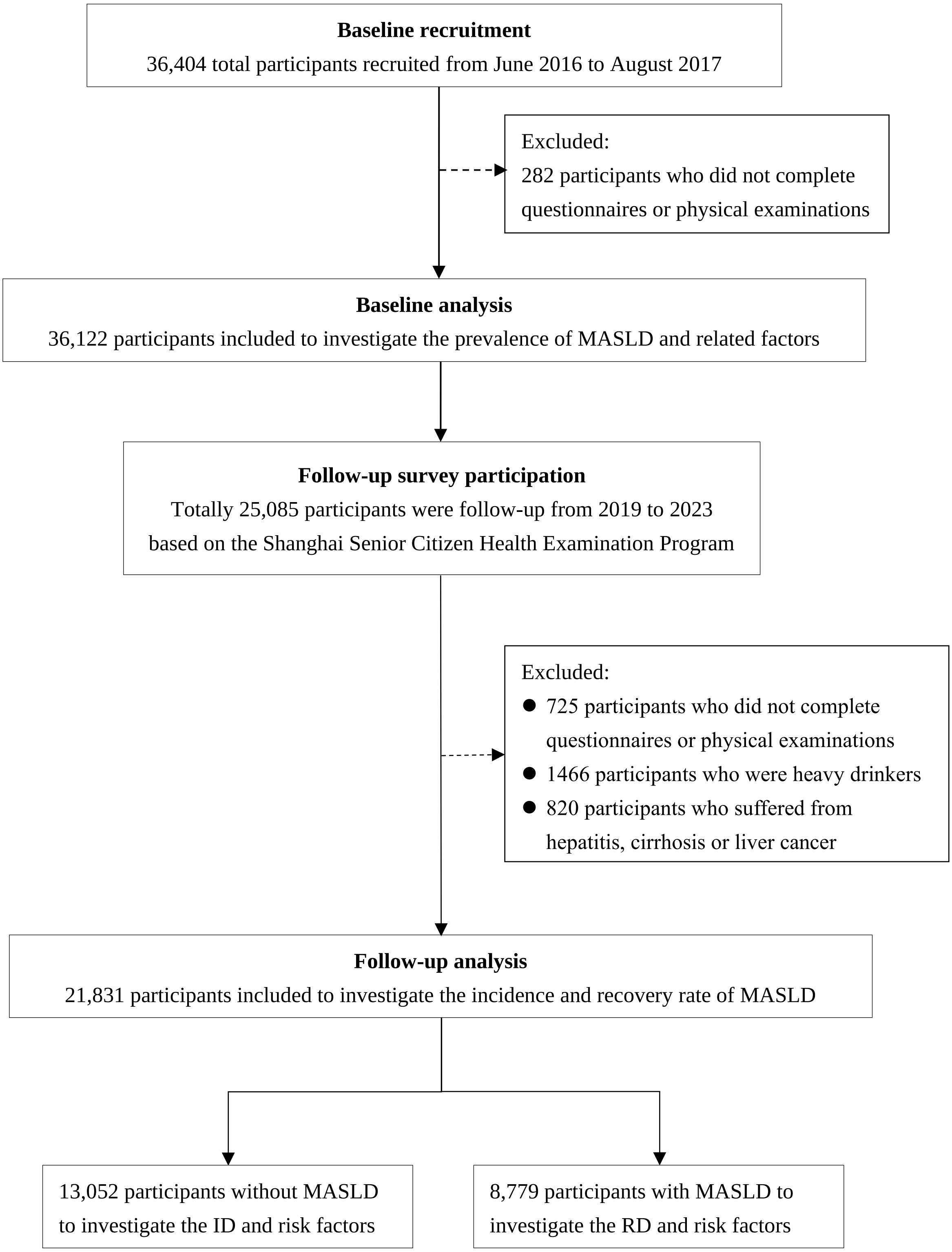


**eFigure1.** Flow chart of the study participants

**eTable 1**. Physical activity types, intensity categories, MET values and codes

| Physical activity type | Intensity | MET | Codes* |
|---|---|---|---|
| Vigorous activity | | 8.0 | |
| Running | Vigorous | 11.4 | Mean of 12010, 12020, 12025, 12027, 12029, 12030, 12040, 12050, 12060, 12070, 12080, 12090, 12100, 12110, 12120, 12130, 12132, 12134, 12135, 12140, 12150, 12170, 12180, 12190 and 12200 |
| Football | Vigorous | 5.6 | Mean of 15210, 15230, 15232 and 15235 |
| Basketball | Vigorous | 7.0 | Mean of 15040, 15050, 15055, 15060, 15070, 15072 and 15075 |
| Tennis | Vigorous | 6.2 | Mean of 15675, 15680, 15685, 15690 and 15695 |
| Boxing | Vigorous | 8.7 | Mean of 15100, 15110 and 15120 |
| Rock climbing | Vigorous | 6.6 | Mean of 15533, 15535, 15537 and 15540 |
| Rope jumping | Vigorous | 11.0 | Mean of 15550, 15551 and 15552 |
| Moderate activity | | 5.3 | |
| Swimming | Moderate | 7.9 | Mean of 18230, 18240, 18250, 18255, 18260, 18265, 18270, 18280, 18290, 18300, 18310, 18320, 18330, 18340 and 18350 |
| Table tennis | Moderate | 4.0 | 15660 |
| Billiards | Moderate | 2.5 | 15080 |
| Volleyball | Moderate | 5.3 | Mean of 15710, 15711, 15720 and 15725 |
| Badminton | Moderate | 6.3 | Mean of 15020 and 15030 |
| Skateboarding | Moderate | 5.5 | Mean of 15580 and 15582 |
| Yoga | Moderate | 2.8 | Mean of 2140, 2150, 2160, 2170 and 2180 |
| Ballroom | Moderate | 6.6 | Mean of 3030, 3038 and 3040 |
| Brisk Walking | Moderate | 4.3 | 17200 |
| Bicycling | Moderate | 8.1 | Mean of 2010, 2011, 2012, 2013, 2014, 2015, 2017 and 2019 |
| Transportation activity | | | |
| Bicycling | Moderate | 6.8 | 1011 |
| Walking | Moderate | 4.0 | 17270 |
| Household activity | | 3.0 | |
| Cooking | Low | 2.8 | Mean of 5035, 5049, 5050 and 5052 |
| Cleaning windows | Low | 3.2 | 5022 |
| Washing clothes | Low | 3.0 | Mean of 5090 and 5092 |
| Sweeping | Low | 3.1 | Mean of 5010, 5011 and 5012 |
| Child care | Low | 2.5 | Mean of 5184, 5185 and 5186 |
| Lifting lightweight items | Low | 3.5 | 5146 |

MET: Metabolic equivalent of tasks.
* Based on the 2011 Compendium of Physical Activities: a second update of codes and MET values. Ainsworth BE, et al. Medicine and Science in Sports and Exercise, 2011;43(8):1575-1581.

**eTable 2.** Prevalence of NAFLD and univariate logistic regression for associations between demographic and behavioral characteristics with NAFLD among community residents in Shanghai, China.

| | Total (N=36,122) | | Male (N=14,671) | | Female (N=21,451) | |
|---|---|---|---|---|---|---|
| | Prevalence, % | OR (95% CI) | Prevalence, % | OR (95% CI) | Prevalence, % | OR (95% CI) |
| Total | 36.8 | - | 33.3 | - | 39.2 | 1.30 (1.24-1.36) |
| Age, years | | | | | | |
| 18~39 | 26.7 | 1 | 40.9 | 1 | 18.2 | 1 |
| 40~49 | 34.1 | 1.45 (1.31-1.60) | 41.6 | 1.21 (1.04-1.41) | 29.7 | 1.92 (1.67-2.20) |
| 50~59 | 42.0 | 2.08 (1.91-2.26) | 38.4 | 1.14 (1.00-1.30) | 44.1 | 3.67 (3.26-4.13) |
| 60~69 | 37.4 | 1.72 (1.58-1.88) | 28.1 | 0.74 (0.65-0.84) | 45.1 | 3.83 (3.39-4.32) |
| ≥70 | 31.0 | 1.28 (1.16-1.43) | 24.0 | 0.54 (0.46-0.64) | 37.1 | 2.73 (2.36-3.15) |
| Education | | | | | | |
| Illiterate or primary school | 38.0 | 1 | 27.9 | 1 | 43.1 | 1 |
| Middle school | 37.5 | 0.97 (0.93-1.02) | 36.5 | 1.42 (1.30-1.54) | 38.3 | 0.81 (0.76-0.87) |
| High school | 33.4 | 0.80 (0.75-0.85) | 37.5 | 1.31 (1.18-1.45) | 29.7 | 0.55 (0.50-0.60) |
| College or above | 25.7 | 0.54 (0.46-0.65) | 35.6 | 1.08 (0.84-1.38) | 18.4 | 0.28 (0.22-0.37) |
| Marital status | | | | | | |
| Married | 37.1 | 1 | 33.6 | 1 | 39.7 | 1 |
| Divorced/ Widowed | 34.1 | 0.88 (0.80-0.97) | 27.2 | 0.79 (0.63-0.98) | 36.2 | 0.87 (0.78-0.97) |
| Single | 26.6 | 0.59 (0.48-0.73) | 31.2 | 0.73 (0.56-0.95) | 20.3 | 0.40 (0.28-0.56) |
| Occupation | | | | | | |
| Officer | 34.3 | 1 | 37.6 | 1 | 30.2 | 1 |
| Professional | 35.6 | 1.05 (0.91-1.22) | 35.4 | 0.90 (0.74-1.09) | 35.8 | 1.30 (1.04-1.62) |
| Worker | 38.0 | 1.17 (1.03-1.33) | 34.8 | 0.93 (0.77-1.11) | 39.9 | 1.53 (1.26-1.85) |
| Farmer | 37.7 | 1.17 (1.03-1.33) | 28.0 | 0.71 (0.59-0.86) | 42.7 | 1.73 (1.43-2.10) |
| Other | 36.2 | 1.09 (0.96-1.23) | 33.8 | 0.89 (0.75-1.06) | 37.8 | 1.40 (1.16-1.69) |
| BMI, kg/m$^2$ | | | | | | |
| <18.5 | 1.4 | 0.07 (0.04-0.12) | 1.8 | 0.13 (0.05-0.31) | 1.2 | 0.05 (0.02-0.10) |
| 18.5~<24.0 | 16.6 | 1 | 12.9 | 1 | 18.8 | 1 |
| 24.0~<28.0 | 49.4 | 5.10 (4.84-5.39) | 42.2 | 5.93 (5.38-6.54) | 55.3 | 5.54 (5.17-5.92) |
| ≥28.0 | 73.6 | 14.35 (13.29-15.50) | 67.0 | 21.18 (18.43-24.35) | 78.5 | 16.32 (14.70-18.12) |
| WC, cm | | | | | | |
| Male <90/Female <85 | 25.8 | 1 | 22.7 | 1 | 27.9 | 1 |
| Male ≥90/Female ≥85 | 64.0 | 5.19 (4.94-5.45) | 59.8 | 6.63 (6.08-7.24) | 66.8 | 5.29 (4.96-5.64) |
| WHR | | | | | | |
| Male ≤0.90/Female ≤0.85 | 21.1 | 1 | 18.8 | 1 | 23.0 | 1 |
| Male >0.90/Female >0.85 | 48.5 | 3.61 (3.42-3.80) | 42.5 | 3.95 (3.62-4.31) | 52.1 | 3.73 (3.49-3.99) |
| Physical activity, MET-h/d | | | | | | |
| <8.0 | 35.4 | 1 | 34.6 | 1 | 36.2 | 1 |
| 8.0~<24.0 | 38.7 | 1.17 (1.12-1.22) | 30.6 | 0.85 (0.78-0.92) | 41.8 | 1.28 (1.21-1.35) |
| 24.0~<40.0 | 39.3 | 1.22 (1.07-1.39) | 24.1 | 0.65 (0.47-0.89) | 43.4 | 1.37 (1.18-1.59) |
| ≥40.0 | 37.0 | 1.10 (0.77-1.57) | 28.2 | 0.69 (0.34-1.43) | 40.6 | 1.27 (0.84-1.91) |
| Sedentary time, h/d | | | | | | |
| 0~4 | 36.2 | 1 | 30.6 | 1 | 39.6 | 1 |
| >4~8 | 37.9 | 1.07 (1.02-1.12) | 36.6 | 1.31 (1.22-1.41) | 39.0 | 0.97 (0.91-1.03) |
| >8 | 38.0 | 1.06 (0.96-1.17) | 40.6 | 1.52 (1.31-1.76) | 35.6 | 0.84 (0.73-0.97) |
| Smoking status | | | | | | |
| Never | 38.8 | 1 | 37.3 | 1 | 39.2 | 1 |
| Former | 30.8 | 0.71 (0.62-0.80) | 30.8 | 0.90 (0.78-1.04) | 25.0 | 0.52 (0.17-1.62) |
| Current | 30.4 | 0.68 (0.65-0.72) | 30.3 | 0.92 (0.86-1.00) | 48.9 | 1.80 (0.99-3.28) |
| Alcohol intake, gram/day | | | | | | |
| 0 | 38.9 | 1 | 38.0 | 1 | 39.3 | 1 |
| 0.1~10.0 | 38.6 | 0.99 (0.89-1.10) | 38.6 | 1.03 (0.92-1.16) | 38.7 | 0.92 (0.62-1.36) |
| 10.1~20.0 | 43.1 | 1.19 (1.01-1.39) | 43.5 | 1.25 (1.06-1.47) | 29.4 | 0.71 (0.25-2.05) |
| 20.1~30.0 | 43.2 | 1.22 (0.97-1.53) | 43.9 | 1.30 (1.03-1.64) | 0 | - |
| Family history of fatty liver | | | | | | |
| No | 36.2 | 1 | 32.7 | 1 | 38.7 | 1 |
| Yes | 48.3 | 1.61 (1.46-1.77) | 48.0 | 1.95 (1.62-2.35) | 48.4 | 1.45 (1.29-1.63) |

BMI, body mass index; MET, metabolic equivalent task; WC, waist circumference; WHR, waist-to-hip ratio.

**eTable 3.** Incidence density of NAFLD and univariate Cox proportional hazard regression of risk factors for NAFLD incidence in Songjiang district.

| Characteristics | Total (N=13,052) | | Male (N=5,015) | | Female (N=8,037) | |
|---|---|---|---|---|---|---|
| | Person-years | HR (95% CI) | Person-years | HR (95% CI) | Person-years | HR (95% CI) |
| Total | 56480.5 | - | 22140.4 | - | 34340.2 | 1.10 (1.03-1.17) |
| Age, years | | | | | | |
| 18~39 | 1586.8 | 1 | 446.3 | 1 | 1140.5 | 1 |
| 40~49 | 2868.2 | 1.13 (0.86-1.48) | 614.4 | 0.83 (0.55-1.26) | 2253.7 | 1.53 (1.06-2.21) |
| 50~59 | 16247.8 | 1.06 (0.84-1.33) | 5026.7 | 0.50 (0.36-0.69) | 11221.1 | 1.69 (1.21-2.34) |
| 60~69 | 27175.9 | 1.70 (1.36-2.13) | 12108.0 | 0.76 (0.55-1.04) | 15067.9 | 2.87 (2.08-3.96) |
| ≥70 | 8601.8 | 1.49 (1.18-1.88) | 3944.9 | 0.68 (0.49-0.94) | 4657.0 | 2.49 (1.78-3.47) |
| Education | | | | | | |
| Illiterate or primary school | 33926.7 | 1 | 11426.8 | 1 | 22499.8 | 1 |
| Middle school | 16476.5 | 0.78 (0.73-0.83) | 7738.0 | 0.96 (0.86-1.07) | 8738.5 | 0.68 (0.62-0.75) |
| High school or above | 6077.4 | 0.72 (0.65-0.80) | 2975.5 | 0.92 (0.79-1.07) | 3101.8 | 0.61 (0.52-0.71) |
| Marital status | | | | | | |
| Married | 51957.0 | 1 | 21096.2 | 1 | 30860.8 | 1 |
| Divorced / Widowed / Single | 4523.5 | 1.03 (0.93-1.15) | 1044.2 | 1.07 (0.85-1.35) | 3479.3 | 0.99 (0.88-1.13) |
| Occupation | | | | | | |
| Officer | 1504.4 | 1 | 871.1 | 1 | 633.2 | 1 |
| Professional | 3681.7 | 1.13 (0.90-1.42) | 2322.5 | 1.12 (0.83-1.52) | 1359.2 | 1.14 (0.80-1.63) |
| Worker | 11316.3 | 1.20 (0.98-1.47) | 4305.6 | 1.30 (0.98-1.71) | 7010.6 | 1.14 (0.84-1.55) |
| Farmer | 16397.4 | 1.46 (1.19-1.79) | 5435.6 | 1.29 (0.98-1.70) | 10961.8 | 1.55 (1.15-2.10) |
| Other | 23580.8 | 1.08 (0.88-1.32) | 9205.5 | 1.01 (0.77-1.32) | 14375.3 | 1.12 (0.83-1.52) |
| BMI, kg/m$^2$ | | | | | | |
| <18.5 | 2132.2 | 0.18 (0.12-0.28) | 605.3 | 0.20 (0.09-0.44) | 1526.9 | 0.17 (0.11-0.28) |
| 18.5~<24.0 | 34567.6 | 1 | 12786.8 | 1 | 21780.8 | 1 |
| 24.0~<28.0 | 16850.1 | 2.40 (2.26-2.56) | 7634.9 | 2.44 (2.20-2.71) | 9215.3 | 2.44 (2.26-2.64) |
| ≥28.0 | 2486.5 | 3.94 (3.56-4.35) | 968.2 | 4.23 (3.58-5.00) | 1518.4 | 3.82 (3.37-4.32) |
| per 1 kg/m$^2$ increase | | 1.18 (1.17-1.19) | | 1.18 (1.17-1.20) | | 1.19 (1.18-1.20) |
| WC, cm | | | | | | |
| Male<90/Female<85 | 46662.4 | 1 | 18865.4 | 1 | 27797.0 | 1 |
| Male≥90/Female≥85 | 9235.7 | 2.41 (2.27-2.57) | 3087.7 | 2.19 (1.96-2.45) | 6148.1 | 2.53 (2.34-2.73) |
| per 1 cm increase | | 1.06 (1.06-1.07) | | 1.07 (1.06-1.07) | | 1.07 (1.06-1.07) |
| WHR | | | | | | |
| Male ≤0.90/Female ≤0.85 | 23352.1 | 1 | 11518.2 | 1 | 11833.9 | 1 |
| Male >0.90/Female >0.85 | 23326.0 | 1.82 (1.70-1.93) | 7472.8 | 1.68 (1.52-1.86) | 15853.2 | 1.90 (1.74-2.06) |
| per 0.05 increase | | 1.27 (1.25-1.30) | | 1.32 (1.27-1.38) | | 1.27 (1.24-1.31) |
| Physical activity, MET-h/d | | | | | | |
| <8.0 | 29466.9 | 1 | 14553.5 | 1 | 14913.4 | 1 |
| 8.0~<24.0 | 24840.0 | 1.01 (0.96-1.08) | 7024.0 | 0.95 (0.86-1.06) | 17816.0 | 1.02 (0.94-1.10) |
| 24.0~40.0 | 1843.4 | 1.04 (0.88-1.22) | 443.3 | 0.98 (0.69-1.39) | 1400.1 | 1.03 (0.86-1.24) |
| ≥40.0 | 187.3 | 0.80 (0.45-1.41) | 49.7 | 1.48 (0.61-3.55) | 137.6 | 0.59 (0.28-1.23) |
| Sedentary time, h/d | | | | | | |
| 0~4 | 38821.9 | 1 | 14173.3 | 1 | 24648.5 | 1 |
| >4~8 | 15365.8 | 1.16 (1.09-1.24) | 6867.0 | 1.25 (1.13-1.39) | 8498.9 | 1.13 (1.04-1.22) |
| >8 | 2155.0 | 0.96 (0.82-1.12) | 1035.2 | 1.13 (0.90-1.41) | 1119.8 | 0.85 (0.68-1.07) |
| Smoking status | | | | | | |
| Never | 43850.7 | 1 | 9560.4 | 1 | 34290.3 | - |
| Former | 2623.3 | 0.71 (0.60-0.85) | 2592.8 | 0.71 (0.59-0.85) | 30.4 | - |
| Current | 10006.6 | 0.85 (0.79-0.92) | 9987.1 | 0.85 (0.77-0.94) | 19.5 | - |
| Alcohol intake, gram/day | | | | | | |
| 0 | 51261.5 | 1 | 17096.2 | 1 | 34165.3 | 1 |
| 0.1~10.0 | 2606.2 | 0.87 (0.76-1.01) | 2483.9 | 0.91 (0.78-1.05) | 122.3 | 1.19 (0.67-2.09) |
| 10.1~20.0 | 1211.3 | 0.86 (0.69-1.06) | 1182.7 | 0.90 (0.72-1.12) | 28.6 | 1.34 (0.43-4.16) |
| 20.1~30.0 | 587.0 | 0.93 (0.68-1.28) | 587.0 | 0.98 (0.72-1.35) | - | - |
| Family history of fatty liver | | | | | | |
| No | 54646.3 | 1 | 21682.0 | 1 | 32964.3 | 1 |
| Yes | 1834.2 | 1.01 (0.85-1.19) | 458.4 | 1.07 (0.77-1.50) | 1834.2 | 0.97 (0.80-1.18) |

BMI, body mass index; CI, confidence interval; ID, incidence density; PYs, person-years; WC, waist circumference; WHR, waist-to-hip ratio.

**eTable 4.** Recovery density of NAFLD and univariate Cox proportional hazard regression of risk factors for NAFLD recovery in Songjiang district.

| Characteristics | Total (N=8,779) | | Male (N=2,947) | | Female (N=5,832) | |
|---|---|---|---|---|---|---|
| | Person-years | HR (95% CI) | Person-years | HR (95% CI) | Person-years | HR (95% CI) |
| Total | 36384.9 | - | 11733.7 | - | 24651.2 | 0.87 (0.81-0.93) |
| Age, years | | | | | | |
| 18~39 | 804.3 | 1 | 453.5 | 1 | 350.8 | 1 |
| 40~49 | 1567.2 | 0.88 (0.67-1.14) | 581.5 | 1.03 (0.70-1.51) | 985.7 | 0.76 (0.52-1.10) |
| 50~59 | 12359.4 | 0.63 (0.50-0.79) | 3759.9 | 0.65 (0.48-0.90) | 8599.4 | 0.58 (0.42-0.80) |
| 60~69 | 17857.7 | 0.98 (0.79-1.22) | 5700.2 | 1.26 (0.93-1.70) | 12157.5 | 0.83 (0.60-1.13) |
| ≥70 | 3796.4 | 1.37 (1.09-1.72) | 1238.6 | 1.89 (1.37-2.61) | 2557.8 | 1.10 (0.79-1.52) |
| Education | | | | | | |
| Illiterate or primary school | 22005.4 | 1 | 4996.3 | 1 | 17009.1 | 1 |
| Middle school | 10651.8 | 0.86 (0.80-0.92) | 4736.4 | 0.75 (0.66-0.84) | 5915.4 | 0.88 (0.80-0.96) |
| High school or above | 3727.7 | 0.83 (0.75-0.93) | 2001.0 | 0.75 (0.64-0.87) | 1726.7 | 0.79 (0.67-0.93) |
| Marital status | | | | | | |
| Married | 34077.2 | 1 | 11281.9 | 1 | 22795.3 | 1 |
| Divorced/ Widowed /Single | 2307.7 | 1.29 (1.15-1.45) | 451.8 | 1.31 (1.02-1.68) | 1855.9 | 1.32 (1.15-1.50) |
| Occupation | | | | | | |
| Officer | 879.3 | 1 | 551.4 | 1 | 327.8 | 1 |
| Professional | 2252.0 | 0.87 (0.68-1.10) | 1368.0 | 1.03 (0.77-1.39) | 884.0 | 0.64 (0.43-0.95) |
| Worker | 7874.2 | 0.92 (0.75-1.14) | 2465.1 | 1.00 (0.76-1.33) | 5409.1 | 0.85 (0.61-1.19) |
| Farmer | 10639.3 | 1.15 (0.94-1.42) | 2489.0 | 1.47 (1.12-1.94) | 8150.3 | 1.02 (0.73-1.41) |
| Other | 14740.1 | 0.99 (0.80-1.21) | 4860.1 | 1.13 (0.86-1.47) | 9879.9 | 0.89 (0.64-1.23) |
| BMI, kg/m$^2$ | | | | | | |
| <18.5 | 27.3 | 2.12 (1.01-4.45) | 5.4 | 6.20 (1.98-19.37) | 21.8 | 1.47 (0.55-3.92) |
| 18.5~<24.0 | 6389.7 | 1 | 1502.6 | 1 | 4887.1 | 1 |
| 24.0~28.0 | 19176.2 | 0.73 (0.67-0.79) | 6620.7 | 0.65 (0.56-0.74) | 12555.4 | 0.74 (0.67-0.81) |
| ≥28.0 | 10449.6 | 0.52 (0.47-0.57) | 3519.8 | 0.43 (0.36-0.51) | 6929.8 | 0.55 (0.49-0.61) |
| per 1 kg/m$^2$ increase | | 0.91 (0.90-0.92) | | 0.89 (0.87-0.91) | | 0.92 (0.90-0.93) |
| WC, cm | | | | | | |
| Male<90/Female<85 | 15858.0 | 1 | 5135.9 | 1 | 10722.1 | 1 |
| Male≥90/Female≥85 | 20142.0 | 0.67 (0.63-0.72) | 6493.9 | 0.64 (0.58-0.72) | 13648.1 | 0.69 (0.64-0.74) |
| per 1 cm increase | | 0.97 (0.97-0.98) | | 0.96 (0.95-0.97) | | 0.97 (0.97-0.98) |
| WHR | | | | | | |
| Male ≤0.90/Female ≤0.85 | 5718.4 | 1 | 2417.7 | 1 | 3300.7 | 1 |
| Male >0.90/Female >0.85 | 24005.4 | 0.74 (0.69-0.81) | 6632.1 | 0.74 (0.66-0.85) | 17373.3 | 0.76 (0.69-0.85) |
| per 0.05 increase | | 0.90 (0.87-0.93) | | 0.85 (0.80-0.90) | | 0.89 (0.86-0.92) |
| Physical activity, MET-h/d | | | | | | |
| <8.0 | 18221.8 | 1 | 8137.8 | 1 | 10084.0 | 1 |
| 8.0~<24.0 | 16594.8 | 0.94 (0.88-1.00) | 3387.0 | 1.05 (0.93-1.18) | 13207.8 | 0.94 (0.86-1.01) |
| 24.0~<40.0 | 1311.8 | 0.78 (0.65-0.94) | 160.9 | 1.13 (0.72-1.75) | 1150.9 | 0.76 (0.62-0.93) |
| ≥40.0 | 131.7 | 0.92 (0.53-1.59) | 28.6 | 0.88 (0.28-2.73) | 103.0 | 0.97 (0.52-1.80) |
| Sedentary time, h/d | | | | | | |
| 0~4 | 23397.2 | 1 | 6509.3 | 1 | 16888.0 | 1 |
| >4~8 | 11378.6 | 0.95 (0.89-1.02) | 4445.8 | 0.88 (0.79-0.99) | 6932.8 | 0.96 (0.88-1.05) |
| >8 | 1508.1 | 0.96 (0.82-1.13) | 762.3 | 0.82 (0.65-1.03) | 745.8 | 1.04 (0.83-1.30) |
| Smoking status | | | | | | |
| Never | 30191.9 | 1 | 5585.0 | 1 | 24606.9 | - |
| Former | 1209.4 | 1.28 (1.08-1.51) | 1203.2 | 1.13 (0.94-1.36) | 6.3 | - |
| Current | 4983.5 | 1.07 (0.98-1.17) | 4945.5 | 0.94 (0.84-1.06) | 38.0 | - |
| Alcohol intake, gram/day | | | | | | |
| 0 | 33458.2 | 1 | 8962.1 | 1 | 24496.1 | - |
| 0.1~10.0 | 1410.5 | 0.93 (0.79-1.10) | 1284.5 | 0.85 (0.72-1.01) | 126.0 | - |
| 10.1~20.0 | 720.9 | 0.95 (0.76-1.19) | 703.7 | 0.81 (0.64-1.03) | 17.2 | - |
| 20.1~30.0 | 326.0 | 1.03 (0.75-1.42) | 326.0 | 0.91 (0.66-1.26) | - | - |
| Family history of fatty liver | | | | | | |
| No | 34288.0 | 1 | 11206.8 | 1 | 23081.2 | 1 |
| Yes | 2096.9 | 0.75 (0.65-0.87) | 526.9 | 0.68 (0.51-0.92) | 1570.0 | 0.79 (0.66-0.94) |

BMI, body mass index; CI, confidence interval; RD, recovery density; WC, waist circumference; WHR, waist-to-hip ratio.
